# Supplementary material for: Present and future ecological niche modeling of garter snake species from the Trans-Mexican Volcanic Belt
Source: PeerJ. 2018 Apr 11;6:e4618. doi: 10.7717/peerj.4618 (PMC5903425; doi:10.7717/peerj.4618)

**Supporting information**

**Table S1.** Percentage of records obtained from fieldwork and online databases.

| % | *T. cyrtopsis* | *T. eques* | *T. melanogaster* | *T. scalaris* | *T. scaliger* |
| --- | --- | --- | --- | --- | --- |
| GBIF | 13.44 | 15.79 | 9.56 | 5.83 | 5.82 |
| iNaturalist | 26.56 | 16.10 | 23.48 | 12.62 | 22.09 |
| Fieldwork | 60 | 68.11 | 66.96 | 81.55 | 72.09 |

**Figure S1**. Graphics of the most important variables for each *Thamnophis* species: (A) *T. cyrtopsis*, (B) *T. eques*, (C) *T. melanogaster*, (D) *T. scalaris* and (E) *T. scaliger*.


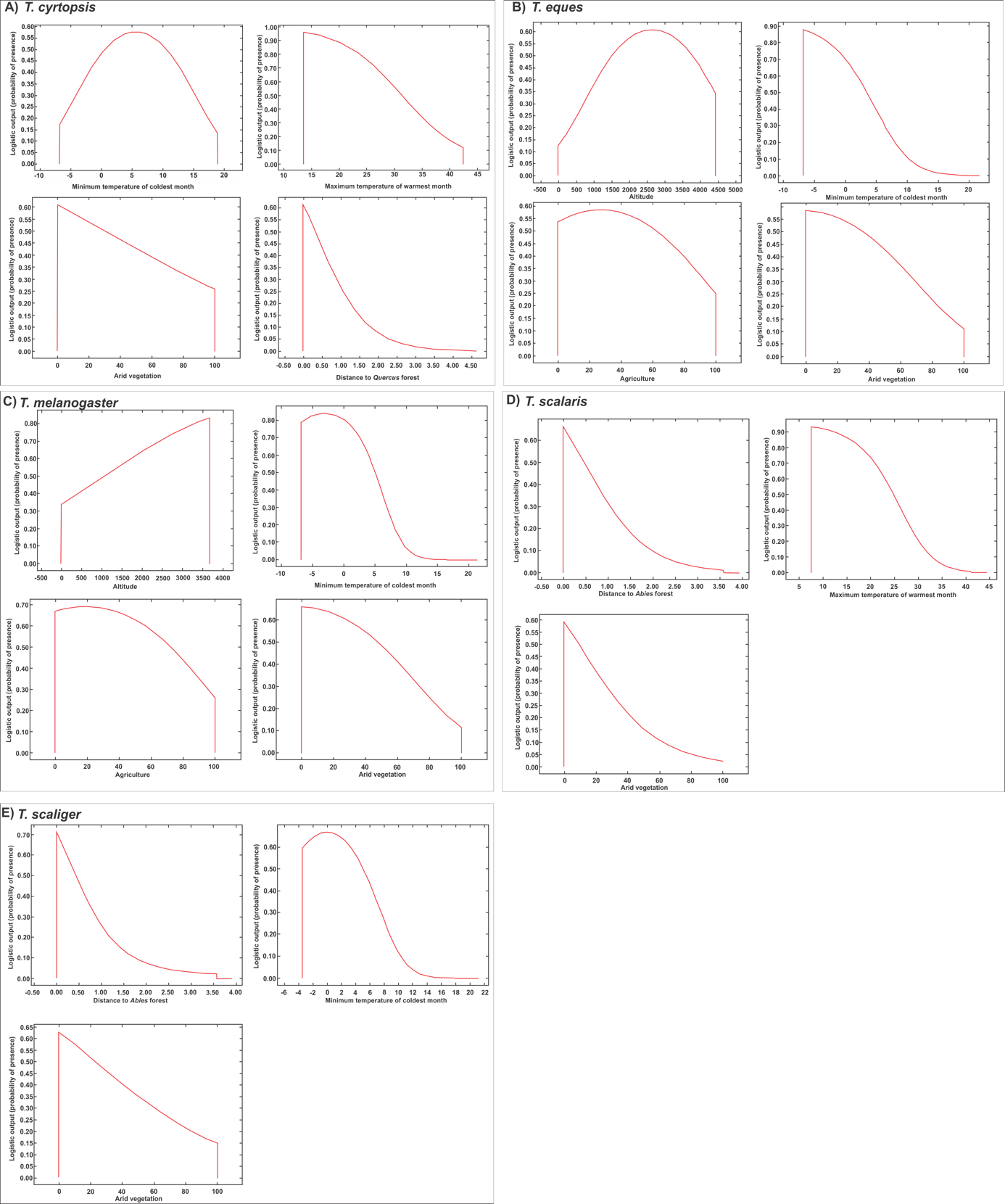


**Figure S2.** Partial-ROC values and graphics; null model (red distribution), distribution of expectations created via bootstrapping replacement of 50% of the total available points and 1,000 resampling replicates (blue distribution).

*T. cyrtopsis*

Mean AUC ratio after 1000 simulations: 1.49695


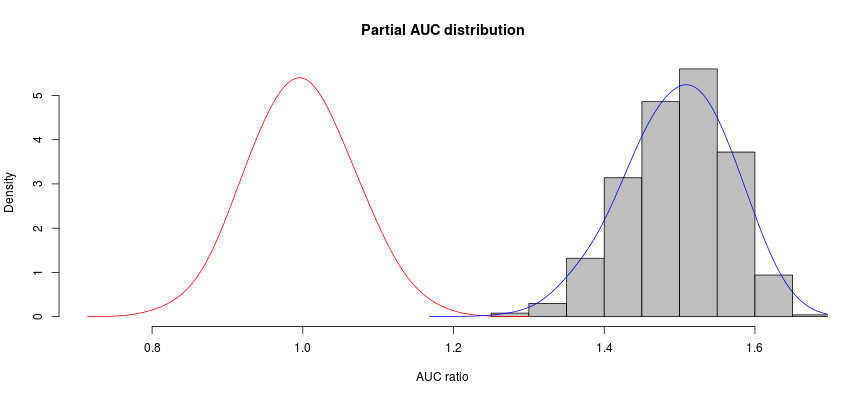


*T. eques*

Mean AUC ratio after 1000 simulations: 1.682837


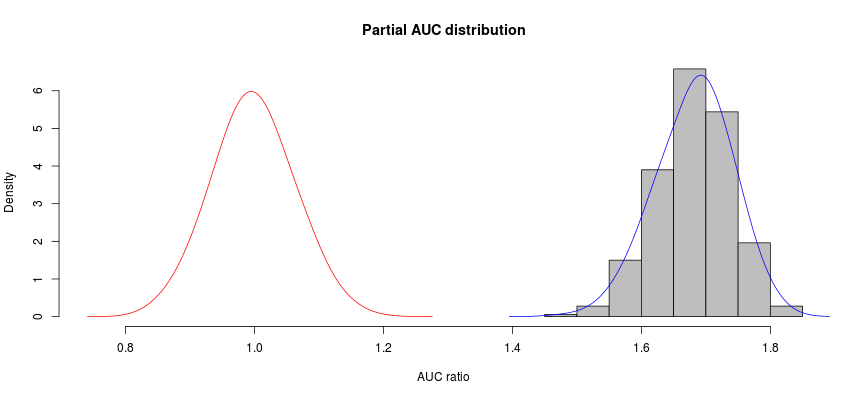


*T. melanogaster*

Mean AUC ratio after 1000 simulations: 1.758803


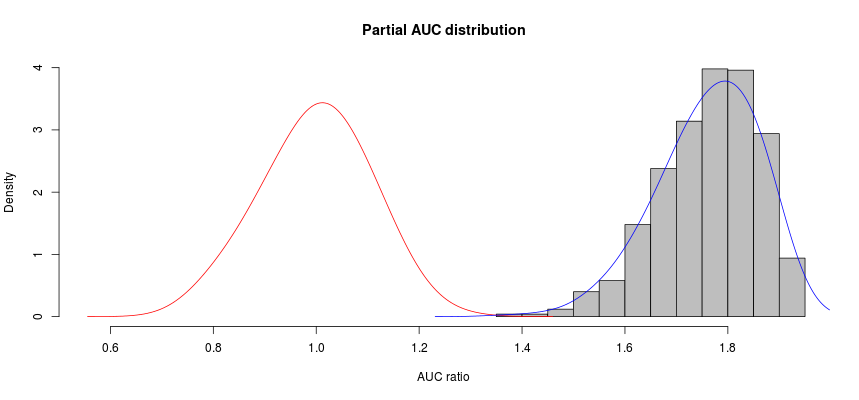


*T. scalaris*

Mean AUC ratio after 1000 simulations: 1.846627


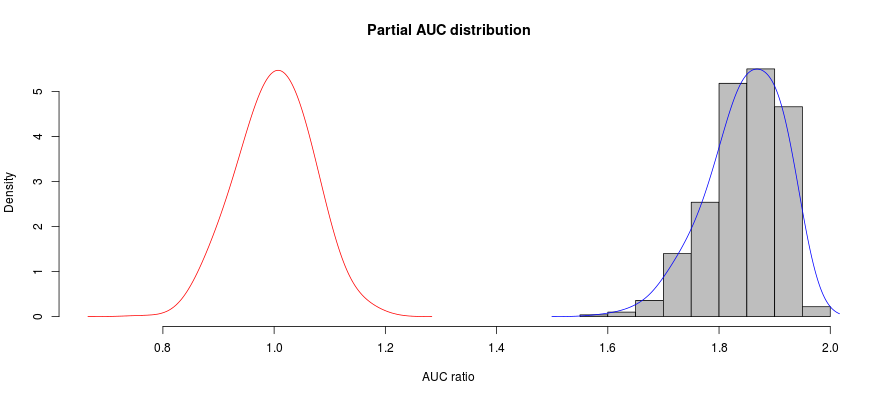


*T. scaliger*

Mean AUC ratio after 1000 simulations: 1.875488


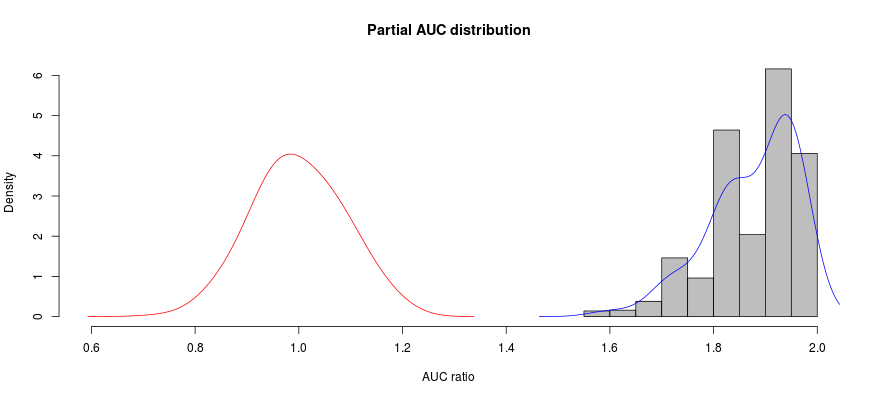

Supplement: Supplemental Information 1 [file peerj-06-4618-s002.docx]
